# Studying on Learning Satisfaction in Teaching Keyboard Courses With Problem-Based Learning Teaching Mode

**DOI:** 10.3389/fpsyg.2022.884311

**Published:** 2022-06-13

**Authors:** Chia-hui Ko

**Affiliations:** Arts and Culture Department, Open University of Kaohsiung, Kaohsiung, Taiwan

**Keywords:** PBL teaching model, teaching effectiveness, self-efficacy, teamwork, keyboard music, adult learning

## Abstract

With the learning characteristics of adults, such as self-directed learning, courses that are preferred in application and practice, this research explores the Problem-Based Learning (PBL) teaching method employed in adult keyboard music learning courses. This research established a research model and investigated the correlation of learning satisfaction with influencing factors such as teaching effectiveness, self-efficacy, and teamwork and verified teaching effectiveness, self-efficacy, and teamwork relationships. Research data has been collected from the keyboard music students of the two classes of the Arts & Culture Department in Open University of Kaohsiung (OUK). Data analysis was conducted in three stages: descriptive statistics, measurement model verification, and structural equation model. The results of the study found that teaching effectiveness has a significant impact on learning satisfaction. Teamwork also has a significant positive impact on learning satisfaction. However, the self-efficacy dimension has little effect on learning satisfaction. Finally, the results of the study found that teaching effectiveness and teamwork both have significant impacts on learning satisfaction. However, the self-efficacy dimension has little effect on learning satisfaction. The student-led presentations went smoothly and the results were quite remarkable, which became a reference for the implementation of other courses for adults in the future.

## Introduction

The trend of an aging society has become a topic that the world must care about. With the advancement of medical care, Taiwan has officially entered an aging society defined by the United Nations as early as 1993. It has entered a senior society in 2018 and will be expected to enter in 2026 for a super-aged society. The Ministry of Education (MOE) in Taiwan (2003) indicated that the elderly increased from 3.43 million in 2018 to 7.15 million in 2065, accounting for 14.5% to 41.2% of the total population growth. The future development directions of White Paper of the education policy for the elderly issued by the MOE in 2006, including (1) Promote the reemployment and career development of the elderly, (2) Cultivate the elderly with the attitude and quality of volunteer service, and (3) Encourage the elderly to return to campus or work as life mentors (Education, 2003). Based on the evolution of Taiwan's population structure, lifelong learning and delayed retirement seem to no longer be slogans. Adult education will inevitably become one of the mainstream of education; and for a free and democratic society to be maintained, it must be emphasized for all people and lifelong learning (Lai et al., 2011). Open University of Kaohsiung (OUK) is based on such an educational philosophy. Since its establishment in 1997, there have been more than 6,000 graduates. The purpose of the school emphasizes an open, free and flexible curriculum structure; the classes include distance learning (non-synchronized) and face-to-face courses. Distance teaching achieves interactive communication and keeps in touch with each other through the school's online platform (ee-class). In addition to arranging the schedule for online lectures, there are four times of face-to-face lectures (once a month) in a semester as a closer communication or the chance of evaluation. And the age of Arts & Culture department students are the oldest among the six ones, with ages ranging from 18 to 80. The students come from all fields of society, and many of them are even graduates from the university; they still want to have dreams coming true and take courses continuously.

A major problem for adults taking keyboard lessons is that students do not feel confident in their own playing. In traditional keyboard lessons, the teacher first explains the music theory and finger techniques, then the teacher demonstrates and then asks the students to practice on their own. Adult students feel frustrated by their slow finger response, especially when they are unable to master two-handed ensemble playing. Problem-Based Learning (PBL) provides a new mode of learning, in which the team works in small groups to identify problems, and after group discussions, share their experiences and solve the problems. Students will feel the benefits of teamwork and enhance their learning effectiveness.

Therefore, the research is based on PBL teaching theory, innovatively applied to keyboard music course teaching, and explores the learning satisfaction of adult learners (over 18 years old) and related influencing factors, verifying that the PBL teaching method is used in adult learning about the effectiveness (Fairchild, 2003) of the keyboard music course. It involves two classes of students participating in teaching practice and research. Moreover, this research adopts a questionnaire survey method to collect learning data to demonstrate the relationship among teaching effectiveness, self-efficacy, team competence, team emotional intelligence, and team interaction. It should provide a reference for course designers and teachers in future adult music ensemble courses.

An adult refers to the role of a socially, psychologically, and economically status in one's own culture and society. Taiwan's current civil law age of majority has been revised down from 20 to 18 years old. With the gradual extension of the average life expectancy in countries around the world, the elderly population has increased year by year; the adult population who participates in lifelong learning in this way is also growing year by year. Laal and Salamati (2012) believe that lifelong learning is to adapt to changes and to enrich and fulfill a natural need of life (Laal and Salamati, 2012).

According to Cercone's theory, adult learning has three main learning characteristics: self-directed learning, experimental learning, and transformational learning (Cercone, 2008). Adult learners are responsible for their own decisions and in many cases also influence others. In addition, with Lippitt et al. (1984) put forward five hypotheses about the characteristics of adult learners: (1)The development of self-concept to become a self-directed learner. (2) Experienced learners. (3) Work development learners. (4) Directional learners. (5) Motivated learners (McGrath, 2009). Although many adults attend classes part-time, adult learners may face the intervention of other factors such as work, family, and economics and interrupt their studies. Furthermore, adult learners prefer learning for practical purposes to learning for academic purposes (Moore and Kearsley, 2011).

Regarding the study of adult learning, Moore and Kearsley (2011) suggested that adult education is an art and science, which covers various types of adult learning, so the life experience of adult learners can be used as a consideration of assessment in addition to knowledge and skills (Yoo and Huang, 2013). At the same time, with Park and Choi re-searched the factors affecting whether adult online learning courses are continued or interrupted. The results proved that personal characteristics (such as age, gender, or education level) do not affect the continuation or interruption of online courses, and external conditions such as the support of the organization, financial issues, and time constraints are the deciding factors that affect whether the course is continued or interrupted. Teachers can design online courses to be more in line with adult learners' use in real life and to reduce the rate of interrupting learning (Park and Choi, 2009). At the Knowles's research pointed out four principles to guide adult learners, including (1) Adult learners must plan and evaluate what they learn; (2) Experience is the basis of learning activities; (3) The mainly learning issue about adult learning is the direct impact on work or personal life; (4) Adult learning is not content-oriented, but problem-solving (McGrath, 2009). Furthermore, with Isenberg (2007) proposed the direction of adult learning, including learning knowledge, learning to do, learning to live, learning to construct, learning to change, and learning to continue. Adult learners are more mature, confident, autonomous, dominant, practical, multi-tasking, purposeful and experienced (Isenberg, 2007).

The age of the students in OUK is widely distributed than traditional universities. There are many people up to 80 years old. Adults, due to their physical and mental maturity, are more sophisticated than the 18–22-year-old students in traditional universities. They have the characteristics of easier communication and cooperation in learning. The motivation of the research is based on Knowles' adult education and learning guidelines, designing a PBL teaching model in OUK group keyboard music course (Lippitt et al., 1984).

## Literature Review and Hypothesis Development

### Problem-Based Learning

PBL is an active problem-oriented group learning model that stimulates learning. In the PBL course teaching model, students have more opportunities to develop their learning skills in a self-directed learning method. Furthermore, the PBL teaching model also guides students to use knowledge to solve problems and take effective self-learning strategies, which is very different from the general traditional teaching (Hmelo and Lin, 2000). PBL has been used since 1969 when the Dutch government ran the Maastricht University Medical School, and it has been still widely employed in various courses (Moust et al., 2005).

The characteristics of the PBL teaching model emphasize that the problem is the starting point of the learning process, and the participants are the center of the learning process. Moreover, PBL is not only a theory, a teaching model but also a practical way of learning; furthermore, the PBL is widely used in different education genres, including language, science, mathematics, etc. (Isenberg, 2007). However, PBL is not used in many music-related courses. Scholar Knowles has studied the application of PBL in music courses. It proved that music can not only release emotions but also enhance learning ability through the connection of emotions and social interaction (Karge et al., 2011).

In addition, the teaching method of PBL Maastricht seven jump process includes seven procedures: (1) Identify the unknown situation and record them. (2) Draft topics for discussion and discuss different views and put them in the topic. (3) Think together, put the known knowledge into the explanation, discuss it, and record it. (4) Review the previous steps, find out possible solutions, and organize them. (5) Draw up the learning goals, reach a consensus among the group members, and the teacher confirms that the group's learning goals are feasible, complete, and appropriate. (6) Each student collects information for learning goals. (7) The group shares the results together; the teacher evaluates the effectiveness and performance of the group learning (Moust et al., 2005). Compared with the traditional curriculum structure, the PBL teaching model encourages participation in the activities of the curriculum, so that students would have a more overall understanding of the learning results.

PBL has a wide range of applied research. For example, the scholar Karge et al. proved that the PBL teaching method is suitable for adult learners. The student's previous knowledge, experience, and ability to study and research can make learning develop into a problem-solving ability (Kolmos et al., 2004). Secondly, the scholar Fink and Krogh's re-search on the PBL teaching model allows students to learn to solve real-life problems with high-end thinking. It is an effective and learner-cored learning model (Dolmans and Schmidt, 2006). In addition, scholars Dolmans and Schmidt studied the influence of the PBL teaching model on cognitive and motivational learning in groups. They found that the group PBL teaching model is conducive to the recall of information, and believed that group discussion has a greater impact on memory than individuals. In addition, even if there are only two or three people in the group, it is still very effective for collaborative knowledge construction. This proves that the PBL group teaching model has a positive effect on self-directed learning (Geitz et al., 2016).

The practice of this research in the keyboard music group class is that after the group discussion, each learning observation record would be written down, including the implementation steps (content), ideas (note any ideas that may solve the problem), facts (note down on the problem known facts), learning topics (note the knowledge and information), action plans (note the method of obtaining information and knowledge), and ultimately, the group ensemble must be completed. Everyone has different ideas and levels, but relying on teamwork, an ensemble piece of about 3–6 min is fully presented, which includes an ensemble of smooth playing at the correct speed and interpretation. Sometimes also needs a moderate music arrangement. Especially, because of the epidemic, starting from the ninth class shift to the online class, the keyboard ensemble of the group is even more challenging. This kind of teamwork learning proves to improve self-efficacy, and it is also an important ability training to ensure that this problem-solving ability can be applied to others (Klassen and Tze, 2014).

### Problem-Oriented Teaching Effectiveness

Teaching effectiveness is the result presented by learners' knowledge, skills, and attitudes after a period of learning time. It also provides a measure of learners' learning outcomes (Andersen, 1979). The scholar Andersen researches the characteristics of teachers to directly predict teaching effectiveness by the influence of attitude, behavior, and cognition and believes that teachers' non-verbal behavior directly affects teachers' teaching effectiveness (Marsh, 1987). Factors that affect the evaluation of teaching effectiveness: including the characteristics of the course (compulsory or electives), course arrangement time, and the degree of difficulty of the course (usually more difficult courses have a higher evaluation) (Klassen and Tze, 2014).

Many studies have pointed out that teachers' psychological characteristics and teaching effectiveness present a positive correlation (McKeachie, 1990). A class with a smaller number tends to have a higher evaluation of teaching (Liu et al., 2006). Scholars Biasutti and Concina believed that an effective teacher includes many personal and professional aspects; the teacher's personal characteristics would affect teaching effectiveness, which is related to achievement orientation or interpersonal relationship orientation, and the behavior presented in the classroom is personal charm or organization. Among them, the teacher's self-efficacy would affect the teaching effectiveness and related to the results of the teaching evaluation (McKeachie, 1990). In addition, the PBL teaching model would promote the learning goals of in-depth content, increase problem-solving skills, and self-directed learning (Dolmans and Schmidt, 2006). According to the research of scholars Liu, Cho, and Schallert, learning in a PBL environment can also promote students' self-efficacy, thereby enhancing teaching effectiveness (Seidel and Shavelson, 2007).

Regarding the research related to problem-oriented teaching effectiveness, scholars Shell, Snow, Frederico, and Montague comprehensively analyzed the results of three types of investigations of teaching effectiveness (Ajai et al., 2013). In addition, scholars Ajai, Imoko, and O'Kwa have done a comparative analysis of PBL teaching and traditional teaching in algebra. The results showed that the PBL teaching model has significantly improved the teaching effectiveness in terms of the important skills of algebra and the depth of learning content (de la Puente Pacheco et al., 2020). Furthermore, the research of scholar de la Puente Pacheco and others found that the PBL teaching model can develop interdisciplinary skills and promote academic performance, and has very good teaching effectiveness (Linnenbrink-Garcia et al., 2011).

### Learning Satisfaction

Learning satisfaction is an important indicator to measure learning achievement. In addition to individual factors of students, learning satisfaction may be affected by teachers, courses, learning environment, and other factors (Boelens et al., 2017). Studies have found that when students are more active, their learning satisfaction is better. Moreover, asking the students questions and doing exercises are all helpful to active learning (Linnenbrink-Garcia et al., 2011). In addition, the psychological characteristics of teachers have long been considered to be highly correlated with teaching effectiveness, and teachers' self-efficacy is also highly correlated with learning satisfaction (Malmia et al., 2019). Then, scholars McGowan and Graham (2009) also found that learning satisfaction with online learning is also the same (Jaggars et al., 2016). Furthermore, teachers require students to provide feedback. This criterion provides learners with knowledge about the results of exercises, which can take many forms, including written or spoken language, to help learners distinguish errors and improve the efficiency of the learning process (Galyen et al., 2010; Malmia et al., 2019).

Learning satisfaction plays a major outcome variable in many studies. For example, in studies by scholars such as Huang, it is believed that teaching quality affects teaching effectiveness, which in turn affects satisfaction. Professional and operational abilities, the use of technology, communication and coordination, the ability to cooperate and self-innovation are all important connotations of teaching quality (Klassen and Tze, 2014). In addition, studies on the relationship between learning satisfaction and social skills in online courses have also proven that students with high social skills have higher learning satisfaction (Bandura, 1997). Furthermore, scholars Boelens, Wever, and Voet also emphasized in the 2017 mixed learning curriculum design to improve learning satisfaction. It is necessary to stimulate students' learning motivation and enhance students' sense of responsibility to complete the work. An effective method is to develop PBL learning and achieve a goal with student group action. Instead of simply teaching in class, it can improve learning efficiency, even learning on the Internet (Van Wart et al., 2019).

In addition, teachers' personality traits are also related to students' learning achievements and teaching implementation. Teachers' teaching effectiveness affects learning satisfaction. From this, it leads to the conclusion that teachers are not born by nature. They can improve teaching effectiveness and promote learning satisfaction. Teachers' teaching effectiveness is more important than personality traits (McKeachie, 1990). Based on the above research, the problem-oriented teaching model would improve teaching efficiency and have a positive impact on learning satisfaction. Therefore, this research infers that the adult learner group uses the PBL teaching model, the teacher's sense of teaching efficacy would have a positive impact on learning satisfaction. There is a positive and significant impact, and the research hypothesis is as:

H1: Students' perception of the teaching effectiveness will positively and significantly affect learning satisfaction in PBL keyboard music courses.

### Self-Efficacy

Self-efficacy is the self-belief that a person can learn or perform a job effectively. According to the scholar's definition of self-efficacy, it refers to an individual's self-belief in what needs to be done (Schunk and Zimmerman, 2012). Emotional and physical state is also an indicator of ability. Scholar Malmia believed that when a positive and desired result is expected, learners are more likely to achieve their goals. When a negative, less expected result is expected, learners want to avoid failure (Galyen et al., 2010). Self-efficacy is an important factor that affects personal learning motivation. At the same time, it also affects personal actions, job choices, continuity, and an effort to achieve the final achievement (Pajares, 2008). In addition, Pajares, 2008 research (2008) mentioned four reasons that influence and enhance self-efficacy: (1) Perfect experience. (2) Alternative experience. (3) Oral or social encouragement. (4) Emotion and physical state (Suryadi and Santoso, 2017). Emotional and physical state is also an indicator of ability.

There are many empirical studies on self-learning effectiveness. For example, ac-cording to the research of scholars Suryadi and Santoso, self-efficacy and adversity quotient are both important factors that positively affect mathematics learning achievement (Bugos and High, 2009). Secondly, scholars Bugos and High (2009) studied the influence of self-efficacy on keyboard music learning, mainly in attitude, achievement, and cooperation (Coutts, 2018). In adult keyboard lessons, choosing the favorite tracks can help improve learner's self-efficacy, self-discipline, opportunities for success, motivation, and overall learning satisfaction (Kurtuldu et al., 2017). Furthermore, in related studies by scholar Bandura and others, it is found that students' learning behaviors are highly correlated by self-efficacy and goal orientation. Generally, people with high levels of self-efficacy are willing to take risks, take actions, and commit to the goals they are pursuing. Furthermore, people with higher self-efficacy are better able to recover from failure and re-establish the situation that they can master and progress (Schunk and Zimmerman, 2012). Self-efficacy can also make goal-oriented work dependent on self-efficacy. When the goal is completed, self-efficacy also improves. Therefore, self-efficacy is related to the effectiveness of job execution and is also positively related to learners' learning satisfaction.

In a 2017 study by scholar Kurtuldu and others on the development of self-efficacy scales in piano lessons, self-efficacy is about basic skills, skill levels, and work guidelines. Self-efficacy is about the perception and application of learning. It is about the level of self-knowledge and self-assessment (Bartimote-Aufflick et al., 2016). In addition, according to Bartimote-Aufflick's (2016) research, student self-efficacy is highly correlated with academic achievement. Self-efficacy is also related to value, self-discipline, metacognition, control trajectory, and application of teaching strategies (Tombaugh and Mayfield, 2014). Based on the above research, self-efficacy would improve teaching effectiveness and has a positive impact on learning satisfaction. Therefore, for learning satisfaction, self-efficacy and teaching effectiveness present a positive correlation. This research infers that adult learners' self-efficacy would have a positive and significant impact on learning satisfaction, and the research hypothesis is as:

H2: Students' self-efficacy will positively and significantly affect learning satisfaction in PBL keyboard music courses.

### Teamwork

According to the definition of Sevens and Campion, no matter how the form of teamwork is carried out, a team should use effective methods to achieve goals. The process of cooperation includes personal and team efforts to communicate, resolve conflicts, make decisions, solve problems, and demonstrate leadership (McKeachie, 1990; Garrison, 2007). Ac-cording to many studies by Garrison et al., social presentation and interaction with peers promoted learning satisfaction (Bolliger, 2004). For online learning activities, learning satisfaction is highly related to the interaction between teachers and students (Dittman et al., 2010).

Team-based classroom collaboration experience challenges both students and instructors (Garrison, 2007). Scholar Sarnikar believed that individuals have innate abilities to achieve team goals in the group. They will construct and develop the process (Stevens and Campion, 1994). In addition, Stevens and Campion believed that the process of teamwork includes personal and cooperative communication, conflict matters, work coordination plans, decision making, problem-solving, and leadership (Murray-Harvey et al., 2005).

Rokhmawati et al. (2016) found that the use of the PBL teaching model improved students' problem-solving skills and self-efficacy. Malmia et al. (2019) believed that creating a healthy and positive interactive environment, helping promote team effectiveness, guiding team construction activities, and developing assignments that can promote mutual learning. Teachers should intervene when needed to promote team-work and process development and provide timely feedback to individuals or teams (Galyen et al., 2010). Moreover, teamwork will increase self-efficacy, thus enhancing learning satisfaction. The effectiveness of promoting teamwork can be analyzed according to three dimensions, which are as follows:

(1) Team competence

According to the research, PBL is considered to be a kind of cross-domain learning to solve problems through group cooperation (Thompson et al., 1996). Team competence is regarded as a kind of team intelligence quotient (Team IQ), which is positively related to team execution. Kakabadse provided evidence of the primary link between team competence and team execution, pointing out that team competence is the key to team execution. Moreover, making teams committed to possessing and using this ability is an important factor in ensuring that the team has a good output value (Willard and Duffrin, 2003). Then, Almond et al. (2009) and Yazici (2005) found that in the classroom, there are many learning and design skills that are conducive to cooperation to enhance the ability of teamwork, including interactive communication skills, conflict resolution skills, coordination skills, and problem-solving skills (Garrison, 2007). Research also showed that the results of teamwork would develop higher-order rational and critical thinking skills (Decker et al., 2009).

(2) Team EQ

Team emotional intelligence quotient (Team EQ) includes the ability of the team to perceive and control emotions for themselves and others (Baruch and Lin, 2012). In situations where the team's emotional intelligence is higher, the team can have better performance and interaction. Having a high EQ in the team would ensure the successful sharing of knowledge, because the high EQ team would be discriminatory and promote mutual understanding, encouraging each other to share the knowledge they have acquired (Bailey et al., 2006; Kauffeld, 2006).

(3) Team interaction

A central and important achievement of the interactive situation is to strengthen communication, avoid conflicts and lead to disasters. In the re-search of Bailey and other scholars, for example, in teamwork, when team interaction is well-established, the team would be more organized and maintained (Gaunt and Treacy, 2020). Regarding the application of teamwork, scholar Gaunt also believed that music ensemble activities in higher education are helping to enhance cooperation (Kokotsaki and Hallam, 2007). Music has also appeared to improve a considerable degree of social and personal skills. The use of amusic ensemble for teamwork can help improve social skills, personal trust, better social adjustment, and positive attitudes (Belland et al., 2009). Teamwork would increase problem-solving skills and self-directed learning, which would also increase learning satisfaction (Hoegl and Proserpio, 2004). The actual contact between teams facilitates a closer observation of progress. Consequently, it is easier for teams to construct start, work content, and results. But if the class is held remotely, the team's coordination activities would be affected (Laffey et al., 2009).

According to the research of Tsai et al. (2008), the social ability is a predictor of student satisfaction with learning. It is also an important factor that directly affects learning satisfaction (such as interaction with peers, sharing of personal resources, etc.). Social navigation, communication skills, and comfortable sharing of resources for online learning affect learning satisfaction (Marks et al., 2005). Based on the above research, team-work would promote social skills. Teamwork and communication would also enhance learning effectiveness, which has a positive impact on learning satisfaction. Therefore, the higher the degree of social interaction among students, the better the teaching effectiveness. This research infers that adult learners know that the effectiveness of teamwork learning would have a positive and significant impact on learning satisfaction through teaching evaluation. The research hypothesis is as:

H3: Students' teamwork will positively and significantly affect learning satisfaction in PBL keyboard music courses.

PBL is a teamwork and student-centered teaching model, so it could enhance students' self-efficacy, thereby promoting learning motivation and enhancing learning satisfaction (Seidel and Shavelson, 2007). The PBL teaching model is widely used in various disciplines, whether it is medicine, nursing, natural science, mathematics, or humanities and music courses, etc. From the research, it can be seen that the effect of implementing PBL courses is higher than the learning satisfaction without PBL (Bartimote-Aufflick et al., 2016). In addition, Galyn, Tsai, and Laffey also pointed out that the implementation of online courses and social skills are also important reasons that affect learning satisfaction. Social skills improved that the group members use learning tools and relationships with others to achieve their learning goals (Bandura, 1997). Moreover, learning satisfaction is highly correlated with the inter-action between peers and mentors (Swan, 2001; Dittman et al., 2010; Lin et al., 2014). From the research, they also found that regardless of men and women, in classroom activities, PBL enhances the competition and cooperation of students with each other. As a result, PBL is also used in another teaching strategy of teachers, thus producing the overall learning effect of students (Bartimote-Aufflick et al., 2016). Based on the above comprehensive research, the research hypothesis is as:

H4: Students' teamwork will enhance students' self-efficacy in PBL keyboard mu-sic courses.

## Materials and Methods

### Research Subjects and Data Collection

The age distribution of adult learners who come to the university is usually 40–70. The age distribution in the Arts and Culture department is even wider. Elderly people over 80 are also very often. The students in the department like the face-to-face courses, and mainly the art and painting courses. Starting from semester 109-1, a face-to-face keyboard group lesson has been added. There are 20 electronic keyboards in a class, and the number of the class is limited to 22 people. It is only two classes in one semester, the afternoon class and the evening class. The keyboard learning courses have been very popular and always fully booked. It explains the needs of the students. Actually, it is more difficult for students to start playing the keyboard than painting the art be-cause the music theory is more abstruse and complicated. The course is still favored that can explain one thing: everyone likes music and likes to make music. Music makes us understand the meaning of a beautiful life. Also, it often has many reasons for adults to take keyboard lessons, and the accomplishment from learning music makes them feel the joy of self-realization.

**Figure 1:**
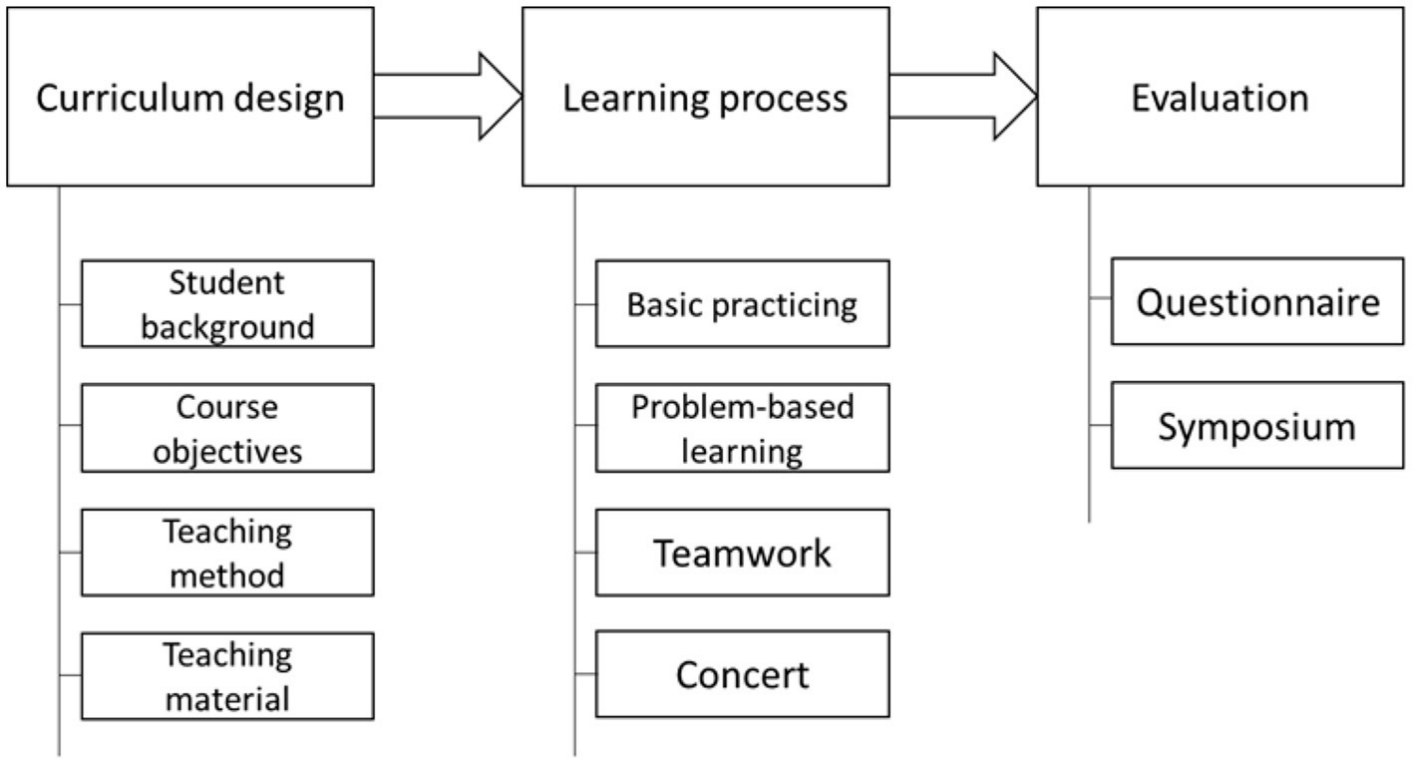
Keyboard music learning course design.

This course is mainly divided into three stages, namely course design, student learning, and learning assessment. The elements considered in the course design include student background, course objectives, teaching methods, and teaching materials. The age of students in this course covers 20–80s. The curriculum objectives are de-signed for basic and practical music theory, proficient keyboard playing principles, ability to fit chords and play songs, keyboard ensemble, music appreciation, etc. The teaching methods include teacher lectures, demonstrations, performances, singing integration, group ensemble, etc. The teaching materials are self-edited teaching materials, traditional teaching materials, individually adapted teaching materials, ensemble teaching materials, etc. In terms of student learning, in addition to basic exercises, it is mainly problem-oriented learning and 4–5 people are formed in groups. Each group is assigned a different keyboard ensemble piece. Through teamwork, questions are raised and discussed with each other. Part of the match and practice, plus the teacher's ad-vice and guidance, complete the ensemble, and at the end, the whole class would hold a music presentation in groups. Finally, the class would evaluate the effectiveness of the course and use a general survey. According to this research model, this study would conduct a questionnaire survey on the research dimension of each student, and then conduct a symposium to discuss the content of the questionnaire and discuss the reasons for the answer as a future course plan. The design and planning content of this course is shown in Figure 1.

In terms of course implementation, there are 12 face-to-face lectures this semester, each for 3.5 h, with four lectures as a unit of instruction step by step, a total of three units. The content of the first unit includes basic music theory, proficient playing skills, and personal repertoire explanation, with the teacher's explanation as to the main teaching activity. From the second unit onwards, PBL learning would be added to the group activities of students, emphasizing teamwork and problem-solving. In the third unit in the second half of the course, the three main repertoires taught and practiced in this course are added. The students choose one of their own, perform the final individual repertoire, and conduct an ensemble presentation, questionnaire survey and symposium in the last class, and a post-meeting party, summing up the course.

Due to the COVID-19 epidemic, the teacher and students experienced a flexible adjustment. The entire content of the third unit has been moved from face-to-face courses to online classes, including online questionnaires, online music presentations, and internet forums. All students completed the equipment structure and software operation. Everyone can be fully equipped in a short time, which also strengthens the PBL teaching model's appropriate application and function.

In this study, the operational definitions of independent variables and dependent variables and the references to the literature are organized in Table 1 below.

**Table 1:** Operational definitions of research variables.

**Research** **variables**	**Operational definitions**	**References**
Learning satisfaction	For me, taking lessons in keyboard music was confident, I would recommend it to others, it was the right decision, it was enjoyable and very satisfying.	Kannan and Narayanan, 2015; Mohammadi, 2015; Joo et al., 2018
Teaching effectiveness	The keyboard music course materials were well organized, the teacher gave many examples and instructions, the pedagogy was innovative, the explanations were clear, the teacher was enthusiastic and the teacher made the course very interesting.	Gibbons et al., 2015
Self- efficacy	When I play the keyboard by myself, I feel comfortable and can mostly follow the instructions with ease.	Baruch and Lin, 2012; Mäntymäki et al., 2014
Team Competence	During the keyboarding course, our team members were able to work together, be compatible and get along well with each other, and were assigned work better than other teams.	Huang et al., 2011
Team EQ	During the keyboarding course, members of our team can fully understand each other's emotions and encourage each other to achieve their goals.	Huang et al., 2011
Team interaction	During the keyboarding course, our team members will help each other, solve problems together, listen to each other, and work together to learn, and have good social interactions.	Fornell and Larcker, 1981; Boelens et al., 2017; Yoo et al., 2018

### Questionnaire Design of Latent Variables

Question items are measured using the Likert seven-point scale, ranging from strongly disagree 1, disagree 2, a little disagree 3, ordinary 4, a little agree 5, agree 6, and strongly agree 7 and other scales are used for scoring. The higher the score, the higher the degree of agreement of the subjects on the research variables. After the questionnaire was designed, experts and scholars were invited to review the questionnaire items and give their opinions. The questionnaire design instructions for each dimension are as follows:

(1) Learning satisfaction

The learning satisfaction dimension of this research refers to the questions used by Joo, So, and Kim in the 2018's study. Their definition of learning satisfaction is the degree of students' satisfaction with Korea Massive Open Online Courses (K-MOOCs). The MOOCs course is a very pleasant experience etc. (Joo et al., 2018). In addition, refers to Mohammadi's 2015 study on the E-learning system, one of the questions can bring confidence to students after learning (Mohammadi, 2015). Concerning Lin, Wang, Lu's 2014 study on consumers' trust in shopping on mobile phones, two questions were used and modified to measure whether the customer strongly recommends others to buy this product and that overall the customer is satisfied with it (Kannan and Narayanan, 2015).

(2) Teaching effectiveness

Regarding the question of teaching effectiveness, Kannan and Narayanan's 2015 research questions on mixed teaching training for teachers (Gibbons et al., 2015) mentioned that the course materials are very organized, and the teachers gave many examples and guidance, and teachers use innovative teaching methods, etc., are all included. And the questions also referred to Gibbons's study on student satisfaction, university rankings, and university applications. The question items are designed as the teacher explained clearly, and the teacher makes the class fun, and the teacher is very enthusiastic in teaching.

(3) Self-efficacy

In terms of self-efficacy, Mäntymäki, Merikivi, Verhagen and Frans's 2014 Questionnaires about Youth Staying in the Virtual World (Mäntymäki et al., 2014), adapted the items to be suit-able for this research situation, such as when I play on my own, I feel comfortable, and I still feel at ease when others are not there to tell me how to play. In addition, Kohnke, Cole, and Bush in 2014 studied family understanding and care of patients and clinicians for medical telemedicine regarding the acceptance level of the equipment, such as I can follow the instructions to complete most of the playing, I can play easily, and I can mostly play the song by myself (Baruch and Lin, 2012).

(4) Teamwork

In the aspect of teamwork, the team competence is used in the 2012 Baruch and Lin competition and virtual team performance study (Huang et al., 2011), including the quality of our team is better than other teams, the amount of work of our team is satisfactory and our planning and allocation are better to other teams, our team members are in the same boat, our team requires inclusiveness and our team gets along with each other.

In the aspect of team EQ, the 2012 Baruch and Lin research question item (Huang et al., 2011) is adopted. For example, our team members have a full understanding of each other's emotions, our team has the ability to be self-aware, our team members can judge emotions from each other's behavior, our team will encourage each other and believe in our own abilities, our team members have the ability to control their emotions and our team work together to achieve the goal.

In the aspect of team interaction, the 2011 Linnenbrink-Garcia, and Rogat, Korkey's emotions in group teaching and the question items in the participation re-search were adopted (Boelens et al., 2017), such as our team helps each other and solves difficulties together, and our team will listen to each other. In 2011 the research question on the process experience of Huang, Chiu, Sung, and Farn in a text interactive environment (Yoo et al., 2018), such as our team showed enthusiasm in the conversation, etc. In 2018, Yooa, Sandersb, and Cerveny's research question related to personnel mobility and psycho-logical ownership were rewritten as I feel teamwork when playing keyboard ensemble, mutual cooperation in keyboard ensemble helps to learn and the keyboard ensemble helps to promote the social interaction of the team (Fornell and Larcker, 1981).

### Data Analysis

The data analysis of this study will be carried out in three stages: descriptive analysis, measurement model verification, and Structural Equation Model. SEM can give a more realistic picture of the original sample information, so the estimation bias is smaller. Again, because it does not do a summed average of scores, the loss of information can be minimized. In general, SEM provides more information and is more rigorous and accurate. Descriptive statistics include statistical analysis of population variables, and calculation of the average and standard deviation of each construct. Understand the concentration degree of each variable and describe it. Then, the study will use the two-step analysis method to measure the instrument and the structure model (Hair et al., 1998). Confirmatory Factor Analysis (CFA) will confirm the reliability and validity of the item, including composite reliability measures the degree of internal consistency of various variables, as well as convergent validity and discriminant validity. In the third stage, the Structural Equation Model (SEM) will be used for analysis to test the fit of the research model, and then to verify the various hypotheses of the research framework. The structural equation model includes path analysis and mediation effect analysis.

**Figure 2:**
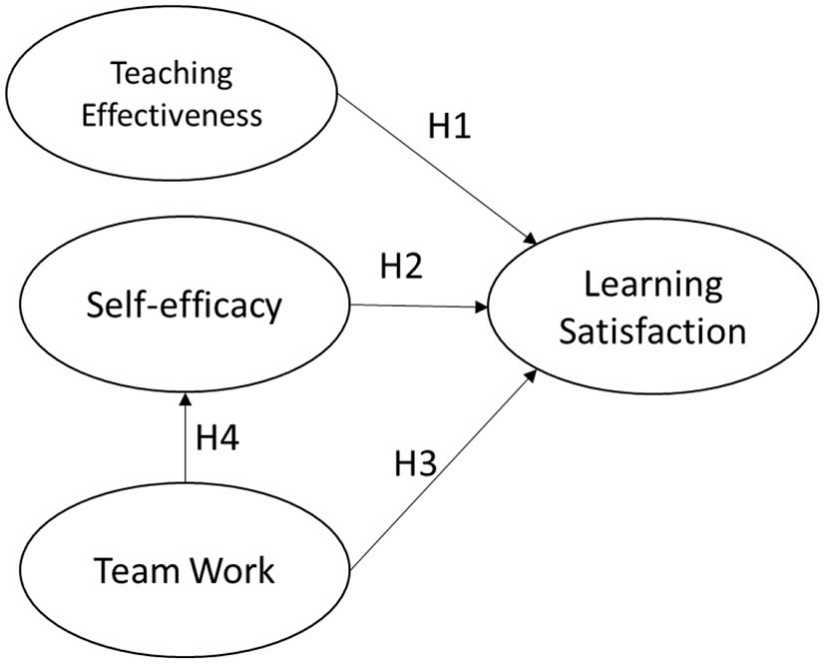
Research model.

The research model is shown in Figure 2.

This study is aimed at students who take keyboard music courses in Open University of Kaohsiung. There are two classes with 20 students in each class. The research content consists of two parts. The first part focuses on the students' basic musical ability: at the beginning of the semester, a musical aptitude test is carried out to obtain the students' basic musical ability data. After the semester, the entire teaching and learning activities are carried out, and the post-test of the music aptitude test is carried out at the end of the semester to understand the learning effectiveness of the students. The second part of the quantitative research, aiming at student learning satisfaction and its influencing factors. This study proposes a research model, including teaching effectiveness, student self-efficacy, and teamwork. Then, a questionnaire was developed to measure the demographic information and research constructs. Collect data by way of census.

The basic demographic variable statistics of students include five items such as gender, age, partner status, occupation, and education level. Moreover, the research variables of this study include learning satisfaction, teaching effectiveness, self-learning effectiveness, teamwork, and sub-constructs of teamwork including team ability, team emotional intelligence, team interaction, etc., to make the concepts be-tween the research variables clearer, explain the scope and design of the following variables one by one:

## Results

### Sample Descriptive Statistics

The basic information of this research includes 6 items [such as gender, age, education level, partner status, occupation (background), class, etc.] (see Table 2). This study used SPSS20.0 statistical software to analyze the average number and standard deviation of the questions, as well as the basic data, including frequency and percent-age of gender, age, partner, education level, class, and occupation.

**Table 2:** Summary table of descriptive statistics.

**Variable**	**Value label**	**Frequency**	**Percent**
Gender	Female	24	70.6
	Male	10	29.4
Age	Under 30 years old	7	20.6
	31–50 years old	10	29.4
	51–70 years old	13	38.2
	71 years old or above	4	11.8
Marital status	Married	22	64.7
	Unmarried	12	35.3
Education	High school or under	8	64.7
	College/University	22	11.8
	Master or above	4	23.5
Occupation	Office worker	7	20.6
	Freelance	6	17.6
	Military, public and educational personnel	6	17.6
	Housewife	2	5.8
	Retire	3	8.8
	Student	5	14.7
	Other	5	14.7

Among them, the majority of respondents are women, with a total of 70.6%. The ages of 31–50 and 51–70 are the largest, accounting for 29.4 and 38.2%, respectively. The students are all adult learners. The largest amount of the education level is in colleges and universities, a total of 22 people, accounting for 47.04%. Most of the students have a partner, a total of 22 people (64.7%). Among related occupations, office workers, freelancers, and military and civilian personnel accounted for the largest number, 20.6, 17.6, and 17.6%. The classes are mainly afternoon classes with 20 students accounting for 58.8%.

### Item Statistical Analysis

The average scores of the constructs are between 6.059 and 6.912, and the highest is 6.912 for teachers who are very enthusiastic about teaching; the second highest is 6.882 for our team to get along well; the third is 6.853 for our team members to help each other in the same boat. The standard deviations are between 0.379 and 1.278, as shown in Table 3.

**Table 3:** Item average and standard deviation analysis table.

**Research variable**	**Item**	**Mean score**	**Standard Deviation score**
Learning satisfaction	1. Keyboard music learning courses give me confidence	6.324	1.065
	2. I strongly recommend others to take keyboard learning courses	6.588	0.857
	3. I think I made the right decision to take the keyboard music learning course	6.706	0.676
	4. The keyboard music learning course is a very pleasant experience for me	6.706	0.719
	5. Overall, I am satisfied with the keyboard music learning course	6.647	0.734
Teaching effectiveness	1. The course materials are very organized	6.588	0.857
	2. The teacher gave a lot of examples and guidance	6.794	0.538
	3. Teachers use innovative teaching methods	6.706	0.719
	4. The teacher explained clearly	6.647	0.849
	5. The teacher makes the class fun	6.765	0.699
	6. The teacher is very enthusiastic in teaching	6.912	0.379
Self-efficacy	1. When I play on my own, I feel comfortable	6.559	0.824
	2. I still feel at ease when others are not there to tell me how to play	6.647	0.774
	3. I can follow the instructions to complete most of the playing	6.441	0.860
	4. I can play easily	6.235	1.046
	5. I can mostly play the song by myself	6.059	1.278
Team Competence	1. Our team members are in the same boat	6.853	0.436
	2. Our team requires inclusiveness and our team gets along with each other	6.824	0.521
	3. Our team gets along well	6.882	0.409
	4. Our planning and allocation are better than other teams	6.559	0.746
	5. The quality of our team's work is better than other teams	6.382	1.015
	6. The amount of work of our team is satisfactory	6.559	0.860
Team EQ	1. Our teams have a full understanding of each other's emotions	6.500	0.826
	2. Our team members can judge emotions from each other's behavior	6.441	0.860
	3. Our team will encourage each other and believe in our own abilities	6.559	0.746
	4. Our team members have the ability to control their emotions	6.618	0.779
	5. Our team works together to achieve the goal	6.529	0.706
	6. Our team has the ability to be self-aware	6.618	0.551
Team interaction	1. Our team helps each other and solves difficulties together	6.647	0.734
	2. Our team will listen to each other	6.676	0.684
	3. Our team showed enthusiasm in the conversation	6.559	0.746
	4. I feel teamwork when playing keyboard ensemble	6.500	0.864
	5. Mutual cooperation in keyboard ensemble helps to learn	6.647	0.734
	6. The keyboard ensemble helps to promote the social interaction of the team	6.647	0.734

### Reliability and Validity of the Construct

Consider the reliability of each item, that is, each item to the extent explained by the latent variable. Hair (1992) suggested that standardized factor loading should be >0.5. The factor loading for all topics in this study is 0.727–0.926. All items have good item reliability. Moreover, Composite Reliability (CR) refers to the consistency of the internal items of the construct. If the CR value of the latent variable is higher, the measurement item is highly correlated. Generally speaking, its value is >0.7 (Hair et al., 2010). The composite reliability of the constructs of this study is higher than 0.929–0.974, indicating that the constructs of this study have good internal consistency. Furthermore, Cronbach's α is a measure of internal consistency. The internal consistency test is suggested >0.7 (Ringle, 2004). In this study, Cronbach's α is 0.905–0.972, which meets the recommended standards and shows good internal consistency.

Average of Variance Extracted (AVE) is the value representing the percentage of latent variables that can be measured by the items. Fornell and Larcker (1981) recommended that the standard should be >0.5. The AVE in this study is 0.678–0.838, which shows that the constructs of this study have good convergence validity, as shown in Table 4.

**Table 4:** Reliability and validity of facets.

**Construct**	**Item**	**Standardized factor loading**	**Cronbach's α**	**Composite reliability (CR)**	**Average of variance extracted (AVE)**
Team Competence (TC)	TC1	0.940	0.958	0.966	0.828
	TC2	0.924			
	TC3	0.920			
	TC4	0.887			
	TC5	0.863			
	TC6	0.922			
Team EQ (EQ)	EQ1	0.902	0.959	0.967	0.829
	EQ2	0.933			
	EQ3	0.931			
	EQ4	0.879			
	EQ5	0.948			
	EQ6	0.868			
Team Interaction (TI)	TI1	0.936	0.946	0.957	0.789
	TI2	0.880			
	TI3	0.787			
	TI4	0.867			
	TI5	0.921			
	TI6	0.931			
Teaching Effectiveness (TE)	TE1	0.952	0.960	0.969	0.8838
	TE2	0.930			
	TE3	0.965			
	TE4	0.931			
	TE5	0.727			
	TE6	0.930			
Self-efficacy (SE)	SE1	0.936	0.905	0.929	0.725
	SE2	0.797			
	SE3	0.776			
	SE4	0.906			
	SE5	0.832			
	SE2	0.797			
Learning Satisfaction (LS)	LS1	0.805	0.947	0.960	0.827
	LS2	0.944			
	LS3	0.912			
	LS4	0.936			
	LS5	0.943			
	LS2	0.944			
Team work(TW)	TC	0.923	0.972	0.974	0.678
	TE	0.905			
	TI	0.909			

### Discriminant Validity

The comparison of cross-loadings and factor loadings for each indicator indicated reasonable discriminant validity, when the factor loading of each scale item for its assigned latent construct is higher than its loading on any other constructs (Hair et al., 2021). Therefore, the constructs in this research had good discriminant validity as Table 5 below.

**Table 5:** Standardized factor loadings and cross loadings of the outer model.

	**EQ**	**LS**	**SE**	**TC**	**TE**	**TI**	**Team work**
EQ1	**0.902**	0.551	0.309	0.709	0.509	0.628	0.816
EQ2	**0.933**	0.510	0.325	0.661	0.459	0.649	0.815
EQ3	**0.931**	0.628	0.469	0.744	0.463	0.733	0.878
EQ4	**0.879**	0.585	0.359	0.699	0.487	0.759	0.850
EQ5	**0.948**	0.474	0.385	0.665	0.311	0.679	0.833
EQ6	**0.868**	0.409	0.359	0.600	0.262	0.580	0.744
LS1	0.544	**0.805**	0.559	0.636	0.532	0.518	0.623
LS2	0.541	**0.944**	0.616	0.805	0.809	0.492	0.681
LS3	0.595	**0.912**	0.653	0.795	0.860	0.515	0.703
LS4	0.512	**0.936**	0.633	0.789	0.674	0.546	0.684
LS5	0.464	**0.943**	0.795	0.754	0.869	0.591	0.668
SE1	0.423	0.738	**0.936**	0.545	0.821	0.572	0.565
SE2	0.369	0.676	**0.797**	0.507	0.802	0.363	0.458
SE3	0.308	0.478	**0.776**	0.276	0.412	0.202	0.290
SE4	0.340	0.590	**0.906**	0.389	0.547	0.558	0.469
SE5	0.252	0.525	**0.832**	0.354	0.476	0.446	0.385
TC1	0.652	0.737	0.420	**0.940**	0.717	0.660	0.830
TC2	0.633	0.785	0.407	**0.924**	0.740	0.651	0.814
TC3	0.651	0.709	0.406	**0.920**	0.639	0.708	0.839
TC4	0.727	0.811	0.482	**0.887**	0.534	0.655	0.835
TC5	0.659	0.679	0.413	**0.863**	0.491	0.604	0.783
TC6	0.754	0.821	0.599	**0.922**	0.613	0.847	0.926
TE1	0.483	0.824	0.678	0.707	**0.952**	0.528	0.633
TE2	0.420	0.768	0.752	0.592	**0.966**	0.360	0.508
TE3	0.412	0.817	0.709	0.631	**0.930**	0.515	0.574
TE4	0.536	0.852	0.655	0.741	**0.965**	0.467	0.644
TE5	0.441	0.778	0.663	0.709	**0.931**	0.440	0.589
TE6	0.129	0.476	0.686	0.263	**0.727**	0.168	0.208
TI1	0.719	0.502	0.497	0.691	0.433	**0.936**	0.853
TI2	0.649	0.341	0.321	0.495	0.297	**0.880**	0.729
TI3	0.686	0.288	0.260	0.485	0.271	**0.787**	0.705
TI4	0.695	0.528	0.523	0.696	0.295	**0.867**	0.823
TI5	0.578	0.664	0.535	0.782	0.531	**0.921**	0.834
TI6	0.630	0.728	0.587	0.847	0.617	**0.931**	0.881

*EQ, Team EQ; LS, Learning Satisfaction; SE, Self-efficacy; TC, Team Competence; TE, Teaching Effectiveness; TI, Team Interaction. The bold values indicate the highest standardized factor loadings*.

### Common Method Variance

Used Harman's One-Factor Test to test the severity of CMV (Podsakoff and Organ, 1986). Exploratory factor analysis for the 37 questions in this study found that the explanatory variance for the first factor was 22.71% and was a non-integrated factor. The impact of CMV was not serious in this study.

### Goodness of Fit

Goodness of Fit (GOF) is a GOF that measures the overall index of the model with 0.1 being weak, 0.25 being moderate, and 0.36 being strong (Vinzi et al., 2010). The GOF of this study is.651, which shows a strong fit.

### Structural Equation Modeling Analysis

**Figure 3:**
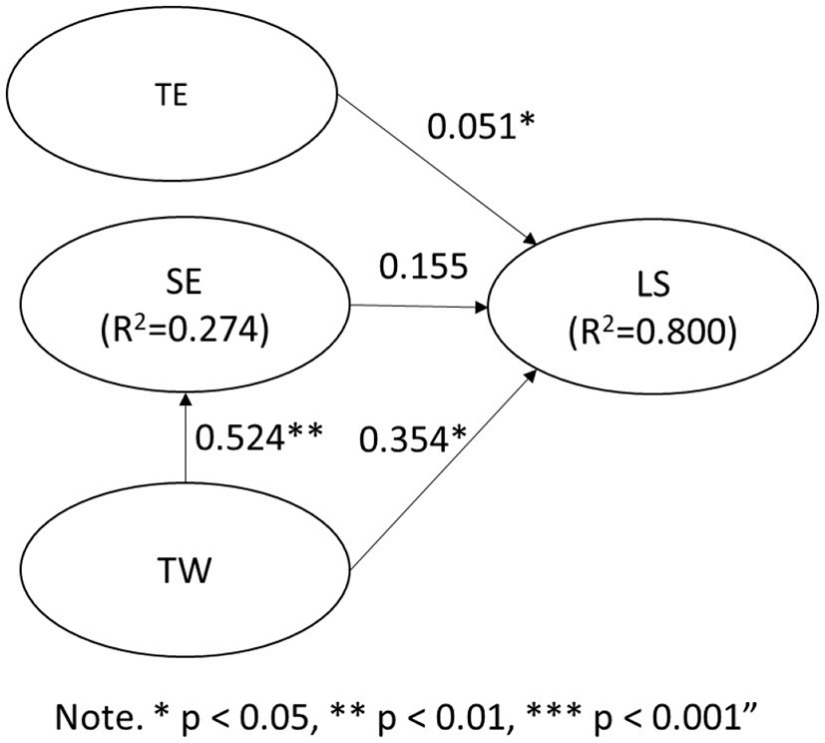
PLS statistical model diagram. LS, Learning Satisfaction; SE, Self efficacy; TE, Teaching; TW, Team Work.

Teaching effectiveness (TE) has a path coefficient of learning satisfaction (LS) of 0.510; standard deviation is 0.246 (*t* = 2.071, *p* = 0.039 < 0.05), so teaching effectiveness (TE) has a significant impact on learning satisfaction (LS). H1 is established. Self-efficacy (SE) has a path coefficient of 0.155 for learning satisfaction (LS); standard deviation is 0.242; (*t* = 0.640, *p* = 0.522 > 0.05), so self-efficacy (SE) has no significant effect on learning satisfaction (LS) H2 invalid. The path coefficient of teamwork on learning satisfaction (LS) is 0.354; standard deviation is 0.168; (*t* = 2.110, *p* = 0.035 < 0.05), so teamwork has a significant impact on learning satisfaction (LS). H3 is established. Teamwork has a self-efficacy (SE) path coefficient of 0.524; the standard deviation of 0.188; (*t* = 2.787, *p* = 0.006 < 0.05), so teamwork has a significant impact on self-efficacy (LS). H4 is established. The results are shown in Table 6 and Figure 3.

**Table 6:** Path coefficients.

**Hypothesis**	**Path** **coefficient** **(β)**	**Standard** **deviation** **(STDEV)**	**T** **statistics** **(|O/STDEV|)**	***P-*** **values**	**2.50%**	**97.50%**
H1: TE → LS	0.510	0.246	2.071	0.039	0.003	0.909
H2: SE → LS	0.155	0.242	0.640	0.522	−0.148	0.762
H3: Team work → LS	0.354	0.168	2.110	0.035	−0.050	0.554
H4: Team work → SE	0.524	0.188	2.787	0.006	0.155	0.863

*LS, Learning Satisfaction; SE, Self-efficacy; TE, Teaching Effectiveness*.

### Explanatory Ability

The *R*^2^ value is the main indicator for judging the quality of the model (Chin, 1998). The *R*^2^ > 0.67 of the latent variable indicates a high explanatory ability, *R*^2^ > 0.33 indicates a moderate explanatory ability, and *R*^2^ > 0.19 indicates a weak explanatory ability (Chin, 1998; Ringle, 2004). According to the analysis results of this study, a self-efficacy (SE) *R*^2^ value of 0.274 has a low to moderate explanatory power; a learning satisfaction (LS) *R*^2^ value of 0.800 has a high explanatory power as shown in Figure 3.

## Conclusions and Discussion

The main part of this research is to introduce PBL teaching methods into adult keyboard music courses. This study verified the learning satisfaction, explored its influencing factors, and used the PBL teaching model as the basis to combine “teaching effectiveness,” “self-efficacy,” and “teamwork” as factors. The research framework and related hypotheses were proposed. After collecting data through questionnaire surveys, the structural equation model was used to test the model and verify the related hypotheses. The PBL learning model is based on problem orientation, focusing more on problem processing and solving, and experiencing the accumulation of knowledge and application. In the past, it was mostly used in clinical medicine learning of medical school, and it is very suitable for adult learners and group learning. Today, the wider application has been very popular in the Open University of Kaohsiung. The PBL learning model is employed for the teaching of adult keyboard lessons in the 2020 academic year. Although people think that it is difficult for adults to learn musical instruments, the results of adult group keyboard lessons are very prominent. This re-search introduced the implication of PBL in musical instrument learning to verify its learning effectiveness, as well as to explore the relationships between related influential variables. The results obtained are as follows:

### Theoretical Contribution

(1) The influence of teaching effectiveness on learning satisfaction

The research results show that the teaching effectiveness of the PBL group key-board course has a significant positive impact on learning satisfaction. This result is similar to the result of the past research (Seidel and Shavelson, 2007). Even in the PBL curriculum, teaching effectiveness is still the main cause of learning satisfaction. In the PBL learning model, teachers also play a very important intermediate role. If there are any problems that cannot be solved during the ensemble of students, teachers still need to intervene and provide assistance in a timely manner. For the prerequisite knowledge about the curriculum, for example, the acquirement of music theory must also be clearly explained and demonstrated. And because the classmates are all adult learners, they have more social experience and mature mentality and can feel the enthusiasm of teachers in teaching. The role of teachers has a very positive impact on teaching satisfaction. At the same time, in the teaching effectiveness aspect of this research, the standard deviation “teachers who are very enthusiastic about teaching” is the highest score among them. The second highest is teamwork, and it can be seen that the role of teachers in teaching effectiveness and teamwork are both important influencing factors of learning satisfaction.

(2) The influence of self-efficacy on learning satisfaction

The results of this research show that the overall average of self-efficacy is the lowest, showing that students are obviously insufficient in self-confidence. When they play the keyboard alone, they still feel difficult. This may be due to a long time impression, “I always find it difficult to play with both hands, and it cannot be achieved without the help of a teacher.” The survey shows that in an ensemble team, the mutual support and guidance of the team members will increase their playing knowledge and skills, which will greatly help the improvement of individual playing ability. It shows that teamwork can improve personal effectiveness. A group ensemble strengthens the communication and confidence between team members. It will make up for the lack of individual self-confidence. In addition, teamwork has a significant positive impact on self-efficacy. This result is similar to the results of previous studies (Ajai et al., 2013; Geitz et al., 2016).

(3) The influence of teamwork on learning satisfaction

The construct of teamwork in this research is divided into three sub-constructs, namely team ability, team emotional intelligence, and team interaction. The keyboard ensemble also strengthens the communication between the team members. Finally, through the research results, it h shows that teamwork has a positive influence on learning satisfaction. This result is similar to the previous research (Macht et al., 2019; Eberz et al., 2020). This shows that every student in the class is very satisfied with teamwork.

Even the face-to-face courses are changed to online courses due to COVID-19, students can still communicate and coordinate with each other through social network media, and work together to complete ensemble works. This is not an easy task for the group, because in addition to taking classes, each student needs to work and take care of their family and other loadings. Besides, they need to find some other time to work on their ensemble tasks. This requires the group to have very consistent goals and only by working together can it be achieved. However, most students still like face-to-face interaction courses. According to the research results, the item “the quality of our team's work is better than other teams” has the lowest score. This also shows that the team's self-confidence is insufficient. This result is consistent with the previous studies (Kim, 2017; Liu and Hou, 2021).

### Implications

(1) Teachers' enthusiasm for teaching

The research results of the importance of teachers' enthusiasm for teaching can be used as a reference for other course designs. Even in student-driven courses by using PBL, the teacher's effectiveness still plays an important role. In addition, because adult learners have the characteristics of independent and experiential learning, teachers must play the role of a good bridge of communication and guidance progressively, which will enhance the effectiveness of learning. In order to increase teachers' enthusiasm for teaching, schools can hold more teaching observation sessions to provide opportunities for teachers to exchange and learn from each other. In short, high teaching effectiveness leads to student satisfaction, which in turn leads to higher enrollment rates and increases the number of students taking courses, as well as their retention rates.

(2) Strengthen the group implementation of group keyboard course

Adult education can be designed to use more teamwork in the curriculum, especially in practical classes. It focuses on practicality and applicability, which is suitable for small group teaching and can also benefit from the PBL learning model. Moreover, teamwork also enhances self-efficacy and personal confidence, which indirectly affects learning satisfaction positively. In addition, the government education department can organize more activities to promote the integration of the PBL learning model into the curriculum, so that students can be familiar with the features and implementation methods of PBL teaching.

(3) PBL can be extended to similar related adult music courses

It is recommended that the PBL learning model be used more often in various mu-sic ensemble courses, not only to increase the fun of practice but also to improve each other's knowledge and practice ability through teamwork and information sharing. There are few cases of adult music ensembles incorporating the PBL learning model, but this study has found that the PBL learning model has a positive impact on learning satisfaction. PBL is not only suitable for problem-solving learning, but also very suitable for group teaching. From the results of this study, if group keyboard lesson is integrated with PBL theory and practice, it is very helpful in improving self-efficacy among learners, especially adult learners. Therefore, in the future, the PBL learning model can be adopted for the implementation of related music ensemble courses, which can increase the interaction between teachers and students and the familiarity between team members, and further enhance learning satisfaction.

### Research Limitations and Future Research

In this study, PBL was implemented mainly for students taking group keyboard courses. In order to verify the satisfaction of course learning, a census of students taking the courses was conducted and the sample size of the study implementation was not large enough. In addition, the education level of the study participants is relatively high, with 74.4% of the study participants having a college or university degree or above. However, the higher the education level, the higher the ability to use information and help each other. It may not be able to fully demonstrate the attitudes and intentions of those with lower education levels. Furthermore, the majority of the study participants were adult learners (79.4%), with fewer respondents under the age of 30, which is an area where future research could be further developed and extended. In addition, this study focuses on group keyboard ensembles, and if there are opportunities in the future, we can conduct intentional studies on the use of other types of musical instruments, such as choral, wind, and strings. We can have a better understanding of the use of various types of music ensembles in the future.

## Data Availability Statement

The original contributions presented in the study are included in the article/supplementary materials, further inquiries can be directed to the corresponding author.

## Author Contributions

C-hK: conceptualization, methodology, investigation, formal analysis, resources, writing—original draft, and data curation.

## Conflict of Interest

The author declares that the research was conducted in the absence of any commercial or financial relationships that could be construed as a potential conflict of interest.

## Publisher's Note

All claims expressed in this article are solely those of the authors and do not necessarily represent those of their affiliated organizations, or those of the publisher, the editors and the reviewers. Any product that may be evaluated in this article, or claim that may be made by its manufacturer, is not guaranteed or endorsed by the publisher.

## References

[B1] Ajai J. T. Imoko B. I. O'kwu E. I. (2013). Comparison of the learning effectiveness of problem-based learning (PBL) and conventional method of teaching algebra. J. Educ. 4, 131–135.

[B2] Almond D. Edlund L. Palme M. (2009). Chernobyl's subclinical legacy: prenatal exposure to radioactive fallout and school outcomes in Sweden. Q. J. Econom. 124, 1729–1772. 10.1162/qjec.2009.124.4.1729

[B3] Andersen J. F. (1979). Teacher immediacy as a predictor of teaching effectiveness. Ann. Int. Commun. Assoc. 3, 543–559. 10.1080/23808985.1979.11923782

[B4] Bailey N. Housley W. Belcher P. (2006). Navigation, interaction and bridge team work. Sociol. Rev. 54, 342–362. 10.1111/j.1467-954X.2006.00617.x

[B5] Bandura A. (1997). The anatomy of stages of change. Am. J. Health Promotion 12, 8–10. 10.4278/0890-1171-12.1.8 10170438

[B6] Bartimote-Aufflick K. Bridgeman A. Walker R. Sharma M. Smith L. (2016). The study, evaluation, and improvement of university student self-efficacy. Stud. High. Educ. 41, 1918–1942. 10.1080/03075079.2014.999319

[B7] Baruch Y. Lin C.-P. (2012). All for one, one for all: Coopetition and virtual team performance. J. Technol. Forecasting Soc. Change 79, 1155–1168. 10.1016/j.techfore.2012.01.008

[B8] Belland B. R. French B. F. Ertmer P. A. (2009). Validity and problem-based learning research: a review of instruments used to assess intended learning outcomes. Interdiscipl. J. Problem Based Learn. 3, 59. 10.7771/1541-5015.1059

[B9] Boelens R. De Wever B. Voet M. (2017). Four key challenges to the design of blended learning: a systematic literature review. Educ. Res. Rev. 22, 1–18. 10.1016/j.edurev.2017.06.001

[B10] Bolliger D. U. (2004). Key factors for determining student satisfaction in online courses. Int. J. E-Learning 3, 61–67.

[B11] Bugos J. A. High L. (2009). Perceived versus actual practice strategy usage by older adult novice piano students. Visions Res. Music Educ. 13, 1–26.

[B12] Cercone K. (2008). Characteristics of adult learners with implications for online learning design. AACE J. 16, 137–159.

[B13] Chin W. W. (1998). The partial least squares approach for structural equation modeling, in Methods For Business Research, ed Marcoulides G. A. (Lawrence Erlbaum Associates Publishers), 295–336.

[B14] Coutts L. (2018). Selecting motivating repertoire for adult piano students: a transformative pedagogical approach. Br. J. Music Educ. 35, 285–299. 10.1017/S0265051718000074

[B15] de la Puente Pacheco M. A. de Oro Aguado C. M. Lugo Arias E. (2020). Understanding the effectiveness of the PBL method in different regional contexts: the case of Colombia. Interactive Learn. Environ. 1–14. 10.1080/10494820.2020.1740745

[B16] Decker B. Landaeta R. E. Kotnour T. G. (2009). Exploring the relationships between emotional intelligence and the use of knowledge transfer methods in the project environment. Knowl. Manage. Res. Prac. 7, 15–36. 10.1057/kmrp.2008.29

[B17] Dittman D. R. Hawkes M. Deokar A. V. Sarnikar S. (2010). Improving virtual team collaboration outcomes through collaboration process structuring. Q. Rev. Distance Educ. 11, 195.

[B18] Dolmans D. H. Schmidt H. G. (2006). What do we know about cognitive and motivational effects of small group tutorials in problem-based learning? Adv. Health Sci. Educ. 11, 321. 10.1007/s10459-006-9012-8 16953462

[B19] Eberz F. Gunkel M. Schlaegel C. Taras V. (2020). A configurational analysis of the effects of EQ and CQ on performance in multicultural teams, in Effects of EQ and CQ on Performance in Multicultural Teams. In Academy of Academy of Management Proceedings (Briarcliff Manor, NY: Academy of Management).

[B20] Education T. M. o. (2003). Toward the Aged Society-the White Book of Policies for Senior Education. Available online at: https://english.ey.gov.tw/Page/61BF20C3E89B856/ed2ff1fb9f2f419d8eb8050710bfd4de

[B21] Fairchild E. E. (2003). Multiple roles of adult learners. New Direct. Student Serv. 2003, 11–16. 10.1002/ss.84

[B22] Fornell C. Larcker D. F. (1981). Structural Equation Models With Unobservable Variables and Measurement Error: Algebra and Statistics. Los Angeles, CA: Sage Publications.

[B23] Galyen K. Tsai I. -C. Laffey J. (2010). The relationship between learning satisfaction and social ability in completely online learning courses, in EdMedia+Innovate Learning [Toronto, ON: Association for the Advancement of Computing in Education (AACE)], 1941–1947.

[B24] Garrison D. R. (2007). Online community of inquiry review: social, cognitive, and teaching presence issues. J. Asynchronous Learn. Netw. 11, 61–72. 10.24059/olj.v11i1.1737 PMC11947623 40151300

[B25] Gaunt H. Treacy D. S. (2020). Ensemble practices in the arts: a reflective matrix to enhance team work and collaborative learning in higher education. Arts Humanities Higher Educ. 19, 419–444. 10.1177/1474022219885791

[B26] Geitz G. Joosten-ten Brinke D. Kirschner P. A. (2016). Changing learning behaviour: self-efficacy and goal orientation in PBL groups in higher education. Int. J. Educ. Res. 75, 146–158. 10.1016/j.ijer.2015.11.001

[B27] Gibbons S. Neumayer E. Perkins R. (2015). Student satisfaction, league tables and university applications: evidence from Britain. J. Econ. Educ. Rev. 48, 148–164. 10.1016/j.econedurev.2015.07.002

[B28] Hair J. (1992). Multivariate Data Analysis with Readings. New York, NY: Macmillan.

[B29] Hair J. F. Anderson R. Tatham R. Black W. (1998). Factor Analysis. Multivariate Data Analysis. Hoboken, NJ: Prentice-Hall, 3, 98–99.

[B30] Hair J. F. Anderson R. E. Babin B. J. Black W. C. (2010). Multivariate Data Analysis: A Global Perspective (Vol. 7).

[B31] Hair J. F. Jr. Hult G. T. M. Ringle C. M. Sarstedt M. (2021). A Primer on Partial Least Squares Structural Equation Modeling (PLS-SEM). London: Sage Publications.

[B32] Hmelo C. E. Lin X. (2000). Becoming self-directed learners: Strategy development in problem based learning, in Problem Based Learning: A Research Perspective on Learning Interactions, eds Eveensen D. H. Hmelo C. E. (Lawrence Erlbaum Publishers), 227–250.

[B33] Hoegl M. Proserpio L. (2004). Team member proximity and teamwork in innovative projects. Res. Policy 33, 1153–1165. 10.1016/j.respol.2004.06.005

[B34] Huang L.-T. Chiu C.-A. Sung K. Farn C.-K. (2011). A comparative study on the flow experience in web-based and text-based interaction environments. J. Cyberpsychol. Behav. Soc. Network. 14, 3–11. 10.1089/cyber.2009.0256 21329437

[B35] Isenberg S. (2007). Applying Andragogical Principles to Internet Learning. Youngs Town, NY: Cambria Press.

[B36] Jaggars S. S. Xu D., and Education. (2016). How do online course design features influence student performance? Comput. Educ. 95, 270–284. 10.1016/j.compedu.2016.01.014

[B37] Joo Y. J. So H. J. Kim N. H. (2018). Examination of relationships among students' self determination, technology acceptance, satisfaction, and continuance intention to use K MOOCs. Comput. Educ. 122, 260–272. 10.1016/j.compedu.2018.01.003

[B38] Kannan K. Narayanan K. (2015). A structural equation modelling approach for massive blended synchronous teacher training. J. Educ. Technol. Soc. 18, 1–15.

[B39] Karge B. D. Phillips K. M. Jessee T. McCabe M. (2011). Effective strategies for engaging adult learners. J. Coll. Teach. Learn. 8, 53–56. 10.19030/tlc.v8i12.6621

[B40] Kauffeld S. (2006). Self-directed work groups and team competence. J. Occup. Org. Psychol. 79, 1–21. 10.1348/096317905X53237

[B41] Kim S. O. (2017). Effects of team based learning on metacogniton, academic achievement, confidence in performance, learning satisfaction. J. Digit. Converg. 15, 361–374. 10.14400/JDC.2017.15.11.361

[B42] Klassen R. M. Tze V. M. (2014a). Teachers' self-efficacy, personality, and teaching effectiveness: a meta-analysis. Educ. Res. Rev. 12, 59–76. 10.1016/j.edurev.2014.06.001

[B43] Kokotsaki D. Hallam S. (2007). Higher education music students' perceptions of the benefits of participative music making. Music Educ. Res. 9, 93–109. 10.1080/14613800601127577

[B44] Kolmos A. Fink F. K. Krogh L. (2004). The Aalborg PBL Model: Progress, Diversity and Challenges. Denmark: Aalborg University Press.

[B45] Kurtuldu M. K. Bulut D., and Practice. (2017). Development of a self-efficacy scale toward piano lessons. Educ. Sci. Theory 17, 835–857.

[B46] Laal M. Salamati P. (2012). Lifelong learning; why do we need it? Procedia Soc. Behav. Sci. 31, 399–403. 10.1016/j.sbspro.2011.12.073

[B47] Laffey J. Tsai I.-C. Amelung C. Hong R.-Y. Galyen K. Goggins S. (2009). The role of social information for social ability, sense of community and satisfaction in online learning, in Annual Conference of American Educational Research Association (San Diego, CA).

[B48] Lai H.-J. Wu M.-L. Li A.-T. (2011). Adults' participation in informal learning activities: key findings from the adult education participation survey in Taiwan. Austral. J. Adult Learn. 51, 409–432.

[B49] Lin J. Wang B. Wang N. Lu Y. J. I. T., and Management. (2014). Understanding the evolution of consumer trust in mobile commerce: a longitudinal study. Information Technol. Manage. 15, 37–49. 10.1007/s10799-013-0172-y

[B50] Linnenbrink-Garcia L. Rogat T. K. Koskey K. L. (2011). Affect and engagement during small group instruction. J. Contemp. Educ. Psychol. 36, 13–24. 10.1016/j.cedpsych.2010.09.001

[B51] Lippitt G. L. Knowles M. S. Knowles M. S. (1984). Andragogy in Action: Applying Modern Principles of Adult Learning. San Francisco, CA: Jossey Bass Publishers.

[B52] Liu M. Cho Y. Schallert D. (2006). Middle school students' self-efficacy, attitudes, and achievement in a computer-enhanced problem-based learning environment. J. Interactive Learn. Res. 17, 225–242.

[B53] Liu Y. M. Hou Y. C. (2021). Effect of multi-disciplinary teaching on learning satisfaction, self confidence level and learning performance in the nursing students. Nurs. Educ. Pract. 55, 103128. 10.1016/j.nepr.2021.103128 34315062

[B54] Macht G. A. Nembhard D. A. Leicht R. M. (2019). Operationalizing emotional intelligence for team performance. Int. J. Ergonom. 71, 57–63. 10.1016/j.ergon.2019.02.007

[B55] Malmia W. Makatita S. H. Lisaholit S. Azwan A. Magfirah I. Tinggapi H.. (2019). Problem-based learning as an effort to improve student learning outcomes. Sci. Technol. Res. 8, 1140–1143.

[B56] Mäntymäki M. Merikivi J. Verhagen T. Feldberg F. Rajala R. (2014). Does a contextualized theory of planned behavior explain why teenagers stay in virtual worlds? Int. J. Inf. Manage. 34, 567–576. 10.1016/j.ijinfomgt.2014.05.003

[B57] Marks R. B. Sibley S. D. Arbaugh J. B. (2005). A structural equation model of predictors for effective online learning. J. Manage. Educ. 29, 531–563. 10.1177/1052562904271199

[B58] Marsh H. W. (1987). The big-fish-little-pond effect on academic self-concept. J. Educ. Psychol. 79, 280. 10.1037/0022-0663.79.3.280

[B59] McGowan W. R. Graham C. R. (2009). Factors contributing to improved teaching performance. Innov. High. Educ. 34, 161–171. 10.1007/s10755-009-9103-6

[B60] McGrath V. (2009). Reviewing the evidence on how adult students learn: an examination of knowles' model of andragogy. Adult Learner Irish J. Adult Commun. Educ. 99, 110.

[B61] McKeachie W. J. (1990). Research on college teaching: the historical background. J. Educ. Psychol. 82, 189. 10.1037/0022-0663.82.2.189

[B62] Mohammadi H. (2015). Investigating users' perspectives on e learning: An integration of TAM and IS success model. Comput. Hum. Behav. 45, 359–374. 10.1016/j.chb.2014.07.044

[B63] Moore M. G. Kearsley G. (2011). Distance Education: A Systems View of Online Learning. Moore, PA: The Pennsylvania State University; Kearsley: University of New England.

[B64] Moust J. H. Berkel H. V. Schmidt H. (2005). Signs of erosion: reflections on three decades of problem-based learning at Maastricht University. Higher Educ. 50, 665–683. 10.1007/s10734-004-6371-z

[B65] Murray-Harvey R. Curtis D. D. Cattley G. Slee P. T., and (2005). Enhancing teacher education students' generic skills through problem-based learning. Teach. Educ. 16, 257–273. 10.1080/10476210500205025

[B66] Pajares F. (2008). Motivational role of self-efficacy beliefs in self-regulated learning, in Motivation and Self-Regulated Learning: Theory, Research, and Applications, eds Schunk D. H. Zimmerman B. J. (Lawrence Erlbaum Publishers), 111–139.

[B67] Park J.-H. Choi H. J. (2009). Factors influencing adult learners' decision to drop out or persist in online learning. J. Educ. Technol. Soc. 12, 207–217.

[B68] Podsakoff P. M. Organ D. W. (1986). Self-reports in organizational research: problems and prospects. J. Manage. 12, 531–544. 10.1177/014920638601200408

[B69] Ringle C. M. (2004). Gütemaße für den Partial-least-squares-Ansatz zur Bestimmung von Kausalmodellen. Univ. Hamburg, Inst. für Industriebetriebslehre und Organisation.

[B70] Rokhmawati J. D. Djatmika E. T. Wardana L. (2016). Implementation of problem based learning model to improve students' problem solving skill and self-efficacy (A study on ix class students of SmpMuhammadiyah). IOSR J. Res. Method Educ. 6, 51–55. Available online at: https://www.iosrjournals.org/iosr-jrme/papers/Vol-6%20Issue-3/Version-4/I0603045155.pdf

[B71] Schunk D. H. Zimmerman B. J. (2012). Motivation and Self-Regulated Learning: Theory, Research, and Applications. Routledge.

[B72] Seidel T. Shavelson R. J. (2007). Teaching effectiveness research in the past decade: the role of theory and research design in disentangling meta-analysis results. Rev. Educ. Res. 77, 454–499. 10.3102/0034654307310317

[B73] Stevens M. J. Campion M. A. (1994). The knowledge, skill, and ability requirements for teamwork: implications for human resource management. J. Manage. Educ. 20, 503–530. 10.1177/014920639402000210

[B74] Suryadi B. Santoso T. I. (2017). Self-efficacy, adversity quotient, and students' achievement in mathematics. Int. Educ. Stud. 10, 12–19. 10.5539/ies.v10n10p12

[B75] Swan K. (2001). Virtual interaction: Design factors affecting student satisfaction and perceived learning in asynchronous online courses. Dist. Educ. 22, 306–331. 10.1080/0158791010220208

[B76] Thompson J. E. Stuart R. Lindsay P.R. (1996), The competence of top team members: A framework for successful performance. J. Manag Psychol. 3, 48–66. 10.1108/02683949610113593

[B77] Tombaugh J. R. Mayfield C. O. (2014). Teams on teams: Using advice from peers to create a more effective student team experience. Acad. Educ. Leadership J. 18, 69.

[B78] Tsai I. C. Kim B. Liu P. J. Goggins S. P. Kumalasari C. Laffey J. M. (2008). Building a model explaining the social nature of online learning. J. Educ. Technol. Soc. 11, 198–215. Available online at: https://anitacrawley.net/Resources/Articles/Building%20a%20model%20explaining%20the%20social%20nature%20of%20online%20learning.pdf

[B79] Van Wart M. Ni A. Rose L. McWeeney T. Worrell R. (2019). A literature review and model of online teaching effectiveness integrating concerns for learning achievement, student satisfaction, faculty satisfaction, and institutional results. Pan Pacific J. Business Res. 10, 1–22.

[B80] Vinzi V. E. Trinchera L. Amato S. (2010). PLS path modeling: from foundations to recent developments and open issues for model assessment and improvement, in Handbook of Partial Least Squares (Berlin; Heidelberg: Springer), 47–82.

[B81] Willard K. Duffrin M. (2003). Utilizing project-based learning and competition to develop student skills and interest in producing quality food items. J. Food Sci. Educ. 2, 69–73. 10.1111/j.1541-4329.2003.tb00031.x

[B82] Yazici H. J. (2005). A study of collaborative learning style and team learning performance. Educ. Train. 47, 216–229. 10.1108/00400910510592257

[B83] Yoo C. W. Sanders G. L. Cerveny R. P. (2018). Exploring the influence of flow and psychological ownership on security education, training and awareness effectiveness and security compliance. J. Decision Support Syst. 108, 107–118. 10.1016/j.dss.2018.02.009

[B84] Yoo S. J. Huang W. D. (2013). Engaging online adult learners in higher education: Motivational factors impacted by gender, age, and prior experiences. J. Cont. High. Educ. 61, 151 164. 10.1080/07377363.2013.836823

